# Experiences of newly qualified midwives during their transition to practice: a systematic review of qualitative research

**DOI:** 10.3389/fmed.2023.1242490

**Published:** 2023-08-16

**Authors:** Jinjin Shi, Xuemei Li, Yongqi Li, Ying Liu, Junying Li, Rongli Zhang, Hui Jiang

**Affiliations:** ^1^Shanghai First Maternity and Infant Hospital, School of Medicine, Tongji University, Shanghai, China; ^2^School of Nursing, Naval Medical University, Shanghai, China; ^3^Department of Obstetrics, Shanghai First Maternity and Infant Hospital, School of Medicine, Tongji University, Shanghai, China; ^4^Department of Nursing, Shanghai First Maternity and Infant Hospital, School of Medicine, Tongji University, Shanghai, China

**Keywords:** newly qualified midwives, transition to practice, experience, meta-synthesis, qualitative systematic review

## Abstract

**Objective:**

To summarize and evaluate the experiences and expectations of newly qualified midwives (NQMs) during their transition from school to clinical practice. One of the main objectives was to provide references for the development of midwifery professional teaching and provide a basis for hospital administrators and instructors of midwifery to develop guidelines and strategies.

**Methods:**

A systemic review of qualitative research using meta-aggregation was conducted. We collected studies from 12 databases between inception and February 2023. All qualitative studies published in English and Chinese that reported on the experiences of NQMs during their transition to practice were included. Two independent reviewers assessed the study quality and the credibility of study findings by using the JBI Qualitative Assessment and Review Instrument. The process of searching followed the Preferred Reporting Items for Systematic Reviews and Meta-Analyses recommendations.

**Results:**

A total of 14 studies were included, and 84 findings were extracted. The results were grouped into 8 new categories and synthesized into 3 main themes: multi-dimensional challenges, physical and emotional responses, and demands and expectations. The included studies were identified to be of good quality and the results of the methodological quality appraisal were all B grade or higher.

**Conclusion:**

The transition period is a critical career development for NQMs. However, they faced various stress during the period, which had a negative impact on their physical and mental health. Therefore, it’s important to deeply understand their challenges and needs. And effective management strategies should be implemented, such as in-depth cooperation between hospitals and schools, improvement of the clinical transition support system, enhancement of continuing education, and standardization of the management system. This may be beneficial to improve the quality of clinical midwifery and maintain the stability and sustainable development of the midwifery team.

## 1. Introduction

Improving the health of mothers and newborns is one of the unfinished Millennium Development Goals and remains a priority in the era of sustainable development goals (1). The Global Strategy for Women, Children, and Adolescents Health (2016–2030) also highlights the significance of ensuring that every woman, child, and adolescent has access to fundamental interventions and a strong team of health professionals (2). Particularly, midwives play a significant role in improving mother–child dyads’ health. Approximately two-thirds of maternal and neonatal deaths can be prevented with the assistance of well-trained midwives (3). However, the State of the World’s Midwifery 2021 shows that only 42 percent of people with midwifery skills work in 73 countries where more than 90 percent of all maternal and newborn deaths and stillbirths occur (4). The survey also reveals that there is a 900,000-midwife deficit worldwide, with a projected 750,000-midwife shortage by 2030 (3, 4).

NQMs represent the future of this profession. However, recruitment and retention of midwives is a major challenge, with a high turnover of NQMs. A previous study indicated that the experiences during the transition to practice had an impact on job satisfaction and employee retention, which was a key factor of the global midwifery shortage (5). The transition period is defined as the period of study and adaptation to work as a registered nurse midwife after completion of a recognized midwifery education program (6). For many newly qualified practitioners, the transition period from students to qualified health professionals is typically 12 to 18 months (7). According to the data from the Royal College of Midwives (RCM), the lack of support of NQMs contributes to attrition ranging from 5 to 10% whereby graduates leave during the first year of practice (8).

To facilitate the retention of valuable midwifery workforce, many countries have developed structured transition support programs to help NQMs successfully transition to practice. However, studies conducted in Australia (7), Canada (9), New Zealand (10), and the United Kingdom (11) reported that NQMs still faced many challenges during the period, including but not limited to increased customer care responsibilities, problems with healthcare systems, political, managerial and role uncertainty (12). These challenges caused them to feel insecure, fearful, and stressed (13). Consequently, the smooth transition into their new roles was interfered and increased personnel losses occurred.

It’s crucial to deeply understand their experiences during the transition period and to explore what factors promote or inhibit the progress. Several qualitative studies have explored the challenges and feelings encountered by NQMs during the transition period but did not provide integrated results. As a result, we conducted a qualitative synthesis that could potentially provide a basis for hospital administrators and instructors of midwifery to develop guidelines and strategies to effectively support NQMs during the transition period.

## 2. Aims

This qualitative systematic review aimed to understand the experiences of NQMs during their transition to practice and to explore factors that promote or inhibit the progress. In particular, the review may provide hospital administrators and instructors of midwifery a new perspective to formulate guidelines and strategies, consequently, it can provide a better training system and platform for NQMs to help them gain fully play their professional roles and positive working experience.

## 3. Methods

### 3.1. Design

A systematic review of qualitative research using meta-aggregation was conducted. The Enhancing Transparency in Reporting the Synthesis of Qualitative Research (ENTREQ) checklist (Supplementary Table S1) was used to report the process and results of synthesis, and enhance transparency (14).

### 3.2. Search strategy

**Figure 1:**
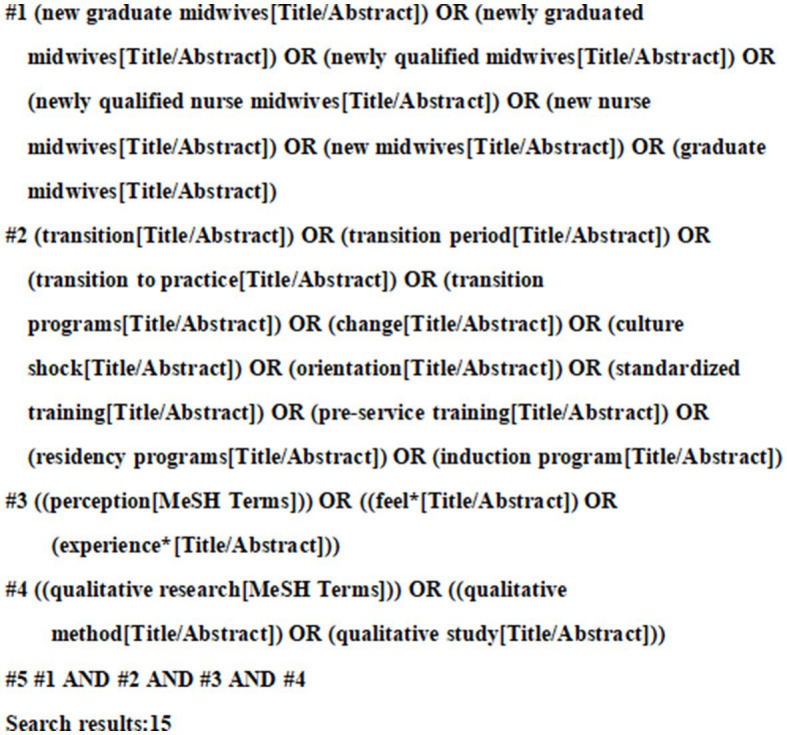
Search strategy in PubMed.

The Preferred Reporting Items for Systematic Reviews and Meta-Analyses (PRISMA) guideline was adopted in this review. A three-step approach was used to identify the studies: (a) an initial limited search via PubMed, (b) a systematic search of electronic databases, and (c) a manual search of journal references. To find search terms, a preliminary limited search via PubMed was first carried out to examine the index words and the derivatives of terms for studies linked to the experiences of NQMs during their transition from education into practice. Then, we systematically searched 12 electronic databases, including eight English language databases: PubMed, Web of Science Core Collection (via ISI Web of Science), MEDLINE (via ISI Web of Science), Cochrane Library, LWW (via OVID), CINAHL Complete (via EBSCO), Scopus, and ScienceDirect, and four Chinese databases: China National Knowledge Infrastructure (CNKI), Wanfang Database (CECDB), VIP Database, and China Biomedical Database (CBM). For different databases, a separate search strategy is designed and optimized based on the corresponding subject terms and search rules. Results were limited to journal articles written in English or Chinese and published before 15 February 2023. The query included five groups of keywords and MeSH terms combined with Boolean operators: (1) (new graduate midwives) OR (newly graduated midwives) OR (newly qualified midwives) OR (newly qualified nurse midwives) OR (new nurse midwives) OR (new midwives) OR (graduate midwives); (2) (transition) OR (transition period) OR (transition to practice) OR (transition programs) OR (change) OR (culture shock) OR (orientation) OR (standardized training) OR (pre-service training) OR (residency programs) OR (induction program); (3) (perception) OR (feel*) OR (experience*); (4) (qualitative research) OR (qualitative method) OR (qualitative study). Finally, the references of each qualifying articles were searched manually to identify further relevant studies. The sample search strategy for PubMed is presented in Figure 1.

### 3.3. Inclusion and exclusion criteria

#### 3.3.1. Inclusion criteria

Studies were included according to the following:

Participant (P): Newly qualified midwives (NQMs) started clinical work for less than three years after graduation.
Interest of phenomena (I): The real experiences of NQMs during their transition from education into practice. The focus was on their stressors, demand, and expectation.
Context (Co): Included studies were those performed during their transition from education into practice.
Study design (S): Qualitative research and mixed-method studies from which the qualitative part could be extracted were included. Studies were included that used any qualitative methodology, including but not limited to phenomenology, grounded theory, case studies, action research, ethnography, and feminist research.

#### 3.3.2. Exclusion criteria

Excluded were studies with qualitative data that were analyzed using quantitative methods; duplicate and unavailable full-text literature; non-English or Chinese literature; research not published in peer-reviewed journals, case reports, conference proceedings, poster abstracts, and theses. Additionally, we looked through their sources to find potential pertinent studies while excluding systematic reviews and other reviews.

### 3.4. Appraisal of methodological quality

By comparing the evaluation criteria of qualitative research, two researchers (JS, XL) who had undergone qualitative research studies and training in evidence-based methods were selected to conduct the study. Two researchers used the “JBI Evidence-Based Quality Evaluation Criteria for Qualitative Studies in Evidence-Based Health Care Centers” for the final independent evaluation of the included studies. Each item is evaluated by “yes,” “no,” “unclear” and “not applicable.” If all 10 items are “yes,” the possibility of bias is minimal and is A. If the above quality criteria are partially met, the possibility of bias is considered to be B. If all items are If “No,” the possibility of bias is considered high as C. After independent evaluation, the results of the two individuals were compared. Third party re-evaluation or arbitration in case of disagreement. The literature with a quality level of C was finally excluded.

### 3.5. Data extraction and synthesis

According to the JBI meta-aggregation, qualitative data were extracted in two steps. Firstly, publication details (author’s name, publication year, country or region, research aim, research design, method of data collection, sampling and data analysis, participants) and findings were extracted. Secondly, verbatim statements about the experiences of NQMs during their transition to practice were extracted for a subsequent meta-synthesize across all included studies. Two reviewers (JS, XL) independently evaluated the plausibility of each finding and identified them into three levels: (1) Unequivocal (U): relates to evidence beyond a reasonable doubt, which may include findings that are matter of fact, directly reported/observed and not open to challenge; (2) Equivocal (E): those that are, albeit interpretations, plausible in light of data and the theoretical framework. They can be logically inferred from the data; (3) Not Supported (NS): when 1 nor 2 apply and when most notable findings are not supported by the data. The extracted findings that had similar meanings were aggregated to form new categories. Eventually, these categories were further synthesized to generate more comprehensive findings, called synthesis findings.

## 4. Results

### 4.1. Search results

**Figure 2:**
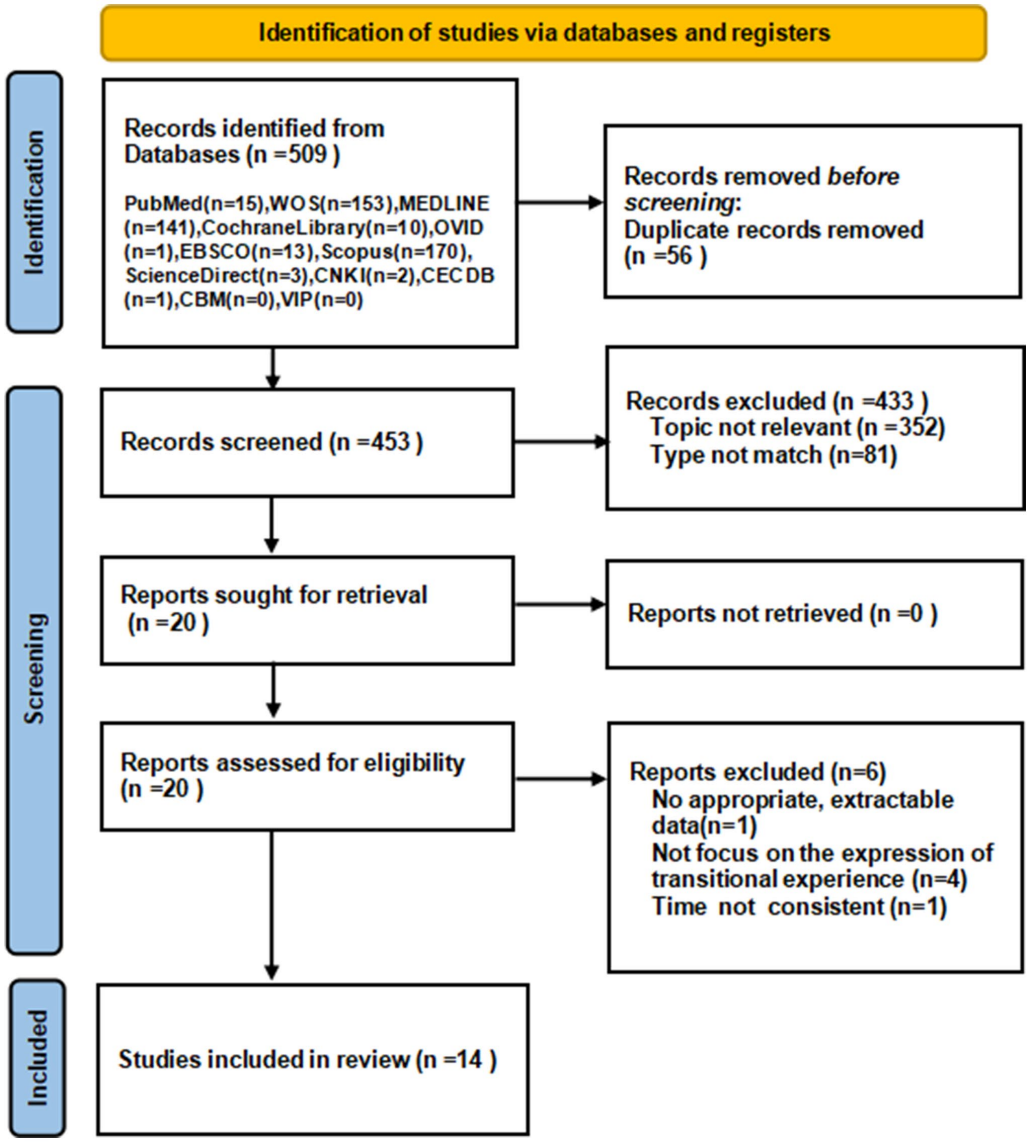
PRISMA flow diagram for article selection.

A total of 509 relevant articles were initially searched from the database. 453 articles were collected in total through NoteExpress after removing duplicates. Two researchers independently read the titles, abstracts and keywords to obtain 20 articles, after reading the full text, 14 articles were included. The detailed search and screening process is showed in Figure 2.

### 4.2. Methodological quality

The quality of the included literature was evaluated and the results were all B grade or higher. The results of the methodological quality appraisal are presented in Table 1.

**Table 1:** Methodological quality of the 14 included studies.

Study^*^	Q 1^**^	2	3	4	5	6	7	8	9	10	Total percent of “yes” (%)
Wier et al. (2022) (15)	U	Y	Y	Y	Y	N	Y	Y	Y	Y	B
Simane-Netshisaulu (2022) (16)	U	Y	Y	Y	Y	N	Y	Y	Y	Y	B
Mtegha et al. (2022) (17)	Y	Y	Y	Y	Y	N	Y	Y	Y	Y	B
Cazzini et al. (2022) (18)	U	Y	Y	Y	Y	N	Y	Y	Y	Y	B
Simane-Netshisaulu et al. (2022) (19)	U	Y	Y	Y	Y	N	Y	Y	Y	Y	B
Donovan et al. (2021) (20)	U	Y	Y	Y	Y	N	N	Y	Y	Y	B
Kool et al. (2020) (21)	U	Y	Y	Y	Y	N	Y	Y	Y	Y	B
Kool et al. (2019) (22)	U	Y	Y	Y	Y	N	Y	Y	Y	Y	B
Norris (2019) (23)	U	Y	Y	Y	Y	N	Y	Y	Y	Y	B
Huang Shu-rong et al. (2017) (24)	U	Y	Y	Y	Y	N	Y	Y	Y	Y	B
Hobbs (2012) (25)	Y	Y	Y	Y	Y	Y	Y	Y	Y	Y	A
Fenwick et al. (2012) (26)	Y	Y	Y	Y	Y	N	Y	Y	Y	Y	B
Clements et al. (2012) (27)	Y	Y	Y	Y	Y	N	Y	Y	Y	Y	B
van der Putten (2008) (28)	Y	Y	Y	Y	Y	Y	Y	Y	Y	Y	A

^*^Critical appraisal (*n* = 10): Y = yes; N = no; U = unclear; NA = not applicable. **Question = Q, Critical appraisal questions for qualitative studies: Q1: Is there congruity between the stated philosophical perspective and the research methodology?; Q2: Is there congruity between the research methodology and the research question or objectives?; Q3: Is there congruity between the research methodology and the methods used to collect data?; Q4: Is there congruity between the research methodology and the representation and analysis of data?; Q5: Is there congruity between the research methodology and the interpretation of results?; Q6: Is there a statement locating the researcher culturally or theoretically?; Q7: Is the influence of the researcher on the research, and vice-versa, addressed?; Q8: Are participants and their opinions adequately represented?; Q9: Are the research ethics according to the current criteria or for recent studies, and is there evidence of ethics approval by an appropriate body?; Q10: Are the conclusions drawn in the research report obtained from the analysis or interpretation of the data?.

### 4.3. Study characteristics

The 14 studies were conducted in the following countries: China (*n* = 1), Malawi (*n* = 1), South Africa (*n* = 2), Netherlands (*n* = 2), Ireland (*n* = 2), the United Kingdom (*n* = 3), and Australia (*n* = 3). These studies involved 238 NQMs. Study designs included qualitative action-research approach (*n* = 1), phenomenological approach (*n* = 4), qualitative descriptive approach (*n* = 5), mix-method study (*n* = 2), ethnography (*n* = 1), and a study described as qualitative without a specific approach (*n* = 1). All the studies were published after 2008 and were original articles. Study characteristics are presented in Table 2.

**Table 2:** Characteristics of the 14 included qualitative studies.

Study	Country	Aim	Research method	No. of participants	Characteristics of participants	Results
Wier et al. (2022) (15)	United Kingdom	To explore the perceptions and experiences of becoming a newly qualified midwifery practitioner	Phenomenological Approach Focus group methodology Purposive sampling Content analysis	8	Newly qualified midwives (NQMs)	Two themes: Becoming a midwife: expectations of self and others Diverse support practices:accessible support and peer support
Simane-Netshisaulu (2022) (16)	South Africa	To explore and describe the experiences of newly qualified midwives with regard to the provision of midwifery services during transition from students to qualified midwives	Qualitative approach with explorative and descriptive Purposive sampling Semi-structured, individual interviews Content analysis	25	Newly qualified midwives who participated in the study were those whose practicing period after completing the training was not more than 12 months and were placed in maternity units in the facilities sampled for the study	Three themes: Excessive workload, resulting in physical exhaustion Roles and responsibilities of newly qualified midwives Collegial relationships: A burden on emotional well-being
Mtegha, Mathews Brave et al. (2022) (17)	Malawi	To explore the transition experiences of newly qualified nurse-midwives working in selected midwifery units in Northern Malawi	Qualitative descriptive approach Purposive sampling In-depth, semi-structured, individual interviews Content analysis	13	Newly qualified nurse-midwives who had completed undergraduate nursing and midwifery education in Malawi (diploma and degree) and had less than two years of transitioning period in practice	Five themes: Theory practice gap. Lack of confidence and skills Inadequate resources Lack of transition support system The workplace conflict
Cazzini et al. (2022) (18)	Ireland	To explore Irish midwives’ experiences of their transition to practice. The objectives were to identify the support required by newly qualified midwives during their first year of clinical practice and to explore what factors facilitate or inhibit newly qualified midwives’ progress during their transition	Qualitative approach Descriptive phenomenology Convenience sampling In-depth, semi-structured interviews Thematic analysis	7	Midwives who commenced their post-registration clinical practice between December 2018 and September 2019	Three themes: Feeling challenged Learning from experience Support
Simane-Netshisaulu et al. (2022) (19)	South Africa	To explore and describe the mentoring process as experienced by newly qualified midwives and experienced mid- wives during the transition period	Qualitative approach with explorative and descriptive Purposive sampling In-depth, semi-structured, individual interviews Thematic analysis	25	Newly qualified midwives working in a maternity unit during their first year of clinical practice following their graduation	Two themes: Newly qualified midwives’ experiences regarding mentoring 1.1. Mentorship: Does it exist? 1.2. Midwifery units: How conducive are they for learning? Experienced midwives’ views regarding mentoring role 2.1. Mentors: How ready are they? 2.2. Shortage of staff and increased workload: How burdensome are they for mentoring?
Donovan,Helen et al. (2021) (20)	Australia	To explore the transition to practice experiences of double degree graduate nurse midwives practicing in either or both nursing and midwifery in the Australian health care system	Husserl’s descriptive phenomenological approach Purposive sampling Semi-structured individual interviews Colizzi’s seven-step phenomenological analysis	23	Participants who were registered with the Australian Nursing and Midwifery Board of Australia and had been employed to work either as a nurse or a midwife or as a dual nurse midwife within the past 9 to 12 months	Four themes: Physical, emotional, and mental exhaustion Safe practice Difficulties in achieving a work–life balance 4. The importance of time to rest and reflect
Kool et al. (2020) (21)	Netherlands	To explore newly qualified Dutch midwives’ perceptions of their job demands and resources during their initiation to hospital-based practice	Qualitative descriptive approach Snowball sampling Semi-structured individual interviews Thematic analysis	21	NQMs who graduated less than three years ago and work as hospital-based midwives in the Netherlands	Four themes: Job demands: high workload, becoming a team member, learning additional medical procedures and job insecurity Job resources: participants experienced the variety of the work, the teamwork, social support, working with women, and employment conditions Personal demands: perfectionism, self-criticism, and fear of failure Personal resources: openness for new experiences, sociability, calmness and accuracy
Kool et al. (2019) (22)	Netherlands	To identify perceived job demands and job resources of newly qualified midwives (NQMs), working in primary midwifery care during their first years in practice	Qualitative descriptive approach Convenience sampling Semi-structured group interviews Thematic analysis	31	NQMs, less than three years after graduation and working in primary midwifery care in the Netherlands	Four themes: Job demands: working as locum; balancing work private life; adjusting to local practice/protocols; dealing with emotions from clients; Administration organizational tasks; colleagues; autonomy Job resources: peers; family; clients; working from home base; textbooks and internet; earning money; colleagues; autonomy Personal demands: perfectionism; prove yourself Personal resources: strict boundaries; flexible; sense of perspective; assertive; self-confidence; humor; persistence
Norris (2019) (23)	United Kingdom	To explore the experience of NQMs in Wales, and to evaluate a current preceptorship program in order to inform the development of a new all Wales preceptorship program	Qualitative action-research approach Convenience sampling Focus group methodology Keep a reflective diary Thematic analysis Spiral analysis	5	NQMs, less than three years after graduation	Five themes: ‘Early days’ ‘A time of transition’ ‘Relationships with colleagues’ ‘Relationships with women’ ‘A new beginning’
Huang Shu-rog et al. (2017)	China	To understand the career experience of new undergraduate midwives	Qualitative approach Purposive sampling In-depth, semi-structured individual interviews Colizzi’s seven-step phenomenological analysis	13	Undergraduate students engaged in midwifery after graduation and entered in July 2015 Clinical work, working time is about half a year; Good communication skills Force, clear language expression; Willing to participate in this study	Five themes: High risk, high intensity, physical and mental stress Full of the joy of new life, have a sense of achievement and value There is a gap between the actual work and the ideal Looking forward to diversified career development See the development potential of the profession and desire to standardize the industry management
Hobbs (2012) (25)	United Kingdom	To ascribe meaning to the everyday experiences of midwives during their first year of practice as they interact with their work environment	Medical ethnographic approach Non-probability sampling Phased interviews Semi-structured interviews Thematic analysis	7	Participants were NQMs, less than three years after graduation	Three themes: “what is a midwife?” Old school midwives (entrenched viewpoint) Service and sacrifice (core/shared dispositions) Being with the woman and making a difference (new ways of thinking)
Fenwick, J et al. (2012) (26)	Australia	To explore the experiences of newly qualified midwives and described the factors that facilitated or constrained their development during the transition from student to registered midwife	Qualitative descriptive approach Convenience sampling Semi-structured interviews Thematic analysis	16	Participants were newly qualified midwives and worked predominantly in standard hospital maternity settings	Four themes: ‘The Pond’: was used to describe new midwives perceptions of the context and culture of hospital-based maternity care The ‘Life-raft’ metaphor was used to describe the importance of midwife-to-midwife relationships The theme of ‘Swimming’ captured the consequence of positive interactions with colleagues and a supportive environment ‘Sinking’ described the consequence of poor relationships with midwives and a difficult working environment
Clements et al. (2012) (27)	Australia	This article reports on newly qualified midwives’ experiences of the core elements of their transition support program	Qualitative descriptive approach Convenience sampling Telephone interviews and focus groups Content analysis	38	Newly qualified midwives from 14 hospitals in the state of New South Wales, Australia	Three themes: The importance of planned clinical rotations and supernumerary time that allowed them to ease into the new clinical area Study days provided an opportunity for graduates to focus on new skills and to connect with their peers Support from colleagues, managers and educators was essential, though workloads often impacted on its availability
van der Putten, Deirdre (2008) (28)	Ireland	To explore newly qualified midwives’ lived experience of clinical practice with a view to gaining a deeper understanding of their individual experiences and as a result, to highlight and inform the practice issues which need to be addressed by midwifery educators	Heideggerian’s descriptive phenomenological approach Purposive sampling Participant observation and in-depth interviews Colizzi’s seven-step phenomenological analysis	6	Newly qualified midwives, all of whom had qualified within the previous six months and who were currently employed within the Maternity Department.	Six themes: Reality shock Feeling prepared Living up to expectations; Theory–practice gap Clinical support and mentorship Continuous professional education

### 4.4. Results of meta-synthesis

The researcher extracted 84 findings from 14 articles and summarized into 8 categories. From the 8 categories, three synthesized findings emerged: multi-dimensional challenges, physical, and emotional responses, and demands and expectations. The main findings with illustrations and levels of credibility are presented in Supplementary Table S2, and the detailed process of synthesis is reported in Supplementary Table S3.

#### 4.4.1. Synthesized finding 1: multi-dimensional challenges

##### 4.4.1.1. Shock from realistic clinical settings

The real work in the delivery room was challenging for NQMs. First, there was a gap between theoretical knowledge in school and clinical practice. Many participants reported that the theoretical knowledge learned in school was relatively outdated or inconsistent with its application in practice. *“Upon reaching the ward, I found that most of the guidelines like HIV guidelines, and some reproductive health standards had changed. There were also new things like CPAP (continuous positive airway pressure). So it was really tough for me as I was referring to old things, yet, the practice had changed on the ground”* (18). In addition, the delivery room was a place of uncertainty, full of challenges and risks directly related to the safety of the mother-infant dyads’ lives. *“Getting a baby into the world alive was what everyone worried about”* (26). At the same time, the lack of human resources was a very serious problem, which led to the huge amount of work that individuals need to carry on. *“…human resource is a challenge…Despite the nursery ward being one of the busy wards, there are times that you are alone on duty and you are expected to do all the activities…”* (17). In addition, the job was insecure and they always faced the possibility of losing their jobs due to the lack of permanent contracts. *“Yes, you know…you have no job security, so you take all the work you can get everywhere…that increases pressure”* (22).

##### 4.4.1.2. High expectations from themselves and others

NQMs were strict with themselves and others also expected more from them. They were eager to prove their abilities quickly, which put a lot of pressure on themselves. *“You want to be the best of the best…I probably put too much pressure on myself…I just need to have confidence and take a deep breath…and I’ll be alright…but then every once I have a little panic…”* (15). They viewed them as true midwives and must be responsible for mothers and babies, so they held themselves to a higher standard. *“you are more independent as a midwife because you have to make more choices you have to have more clinical judgment…more pressure, more responsibility and being more accountable for what I do”* (18). Experienced colleagues also had high expectations of NQMs because they thought that NQMs were fresh out of school and knowledgeable. *“I think are they going to perceive me as: well, you are newly qualified and you need to be able to do this…”* (15). Besides, they were also expected to do more than work, some even beyond their current capabilities. *“After three months I was left in charge of the ward as the only midwife and when I questioned it I was told (by the manager) ‘Oh, you can manage … ‘because you have got experience a nurse’…”* (27).

##### 4.4.1.3. Lack of transitional support

Many participants reacted hospitals did not provide a perfect support system during the transition to practice, which increased the difficulty of adapting to new environments and transitioning into new roles. At first, there was a lack of the training about the hospital-related management system. *“There are a lot of dynamics in the hospital…And it took me some time to realize which disciplines are involved and which agreements are made per hospital, and about protocols. And even if you have a protocol, the usual way of doing things can be different, and it takes a while before you know this…”* (21). Secondly, the absence of training in clinical skills made NQMs scared. *“The situation is not good at all; in some instances, you have to learn through trial and error. I was so scared of resuscitating a new-born baby, until one day in which I had to practice it all by myself”* (16). Thirdly, NQMs were frustrated about the lack of support from experienced mentors. *“Do you know in my whole year as a new grad [graduate] I do not think I worked with an [midwifery] educator once”* (26). Finally, NQMs often did not receive a positive response from colleagues when they asked for help. *“…however, some are unfriendly. The unfriendly ones give bad and demotivating remarks when we seek for assistance. It’s bad”* (19). *“…fter you have had handover and they are like, ‘Oh fine, do not worry.’ Then they go to the desk and they are like: ‘I do not want to come on to work after her, she leaves everything for the night staff”* (23).

#### 4.4.2. Synthesized finding 2: physical and emotional responses

##### 4.4.2.1. Physical fatigue

NQMs suffered physically fatigue because of the high intensity of work. And due to the shift system, their life was irregular and did not get enough rest, which even affected their safe operation. *“It’s just exhausting, just physically. Some days you just need to sleep”* (20). *“I do not think I will ever get used to shift work! It’s almost debilitating…you just start to doubt yourself and I think ‘Am I safe practicing when I’m this tired or this exhausted?’”* (20).

##### 4.4.2.2. Negative emotion: lack of confidence, fear, and loneliness

NQMs’ negative feelings included lack of confidence, fear, and loneliness. When they entered a new environment, due to unfamiliar with the environment and lack of training, they were not confident in themselves. *“When we were students, we were never given any chance to practice managing the unit, but suddenly you are expected to manage the unit including patients, staff members, equipment and supplies. This is not easy. Especially because you do not feel confident enough to delegate duties to some members of staff”* (16). In addition, they often felt fear when facing some clinical problems alone. *“I was absolutely terrified just because I had not done it for so long…and I would be like, I do not know if I can do this. I do not know what I’m doing”* (20). For many NQMs who work away from home, they were not accompanied by family and friends and felt very lonely. *“Just the loneliness was probably the most emotionally draining thing”* (20). It is very important for them to have time to spend time with their family and friends, and to get their support and company. *“It’s really important that you are able to debrief with friends and family because you will say things to friends and family that you would not say to work colleagues”* (20).

#### 4.4.3. Synthesized finding 3: demands and expectations

##### 4.4.3.1. Support from peers, colleagues, and managers

NQMs desperately needed substantial support from peers, colleagues and managers, which was like a light in the dark, making them less nervous and more confident. Support from peers reduced their anxiety. *“[having peer support] should be part of the support process…an opportunity for us to feel like our concerns are being listened too…It’s not just us talking amongst ourselves…”* (15). Support from colleagues, especially experienced midwives, helped them smoothly transition, which was essential for them to adapt quickly to their new roles. *“…they orientate you, they explain everything to you, the routine, the procedures and practices and they still keep an eye on you, you know make sure you are doing ok and that gives you confidence”* (28). In addition, it was important to have an approachable leader who can provide great clinical and emotional support to NQMs. *“The manager on the ward was excellent, she was always checking in with you making sure that you were doing okay”* (18).

##### 4.4.3.2. Improve professional competence

NQMs wanted to improve their professional skills, including clinical decision-making ability, humanistic care, and clinical professional skills. First, NQMs expected independent clinical decision-making capabilities and they needed to have independent autonomy in the care of their patients. *“I also dared to make decisions and I dared to pick up [tasks] independently and it is really not that I needed help with anything and everything. I think that I can generally work independently”* (16). Second, almost all the participants hoped to give more humanistic care to women. *“My frustration is mainly to do with the women not getting the care that maybe they expected or I expected them to get”* (25). *“For me, being with the woman is just a part of my soul…but I do not get a lot of time to do that…I have to do a lot of things rather than actually being with woman…”* (18). By improving humanistic care, in turn inspires them to work better. *“When I support a woman…that is why I chose this profession. Then it is easy to get out of my bed in the night. Moreover, I feel that my work is my passion, and my passion is my work”* (22). Finally, NQMs would like to receive more professional training or study in order to adapt faster to the new environment and further strengthen their professional skills. *“I hope to continue my study in midwifery and continuously improve my skills in technical operation and clinical thinking”* (29).

##### 4.4.3.3. Standardize the management system

NQMs desired to standardize the industry management and establish an independent midwifery management and training system. *“For young midwives, there should be a standardized training system, and they should have standardized training just like clinicians. After all, this line of work requires a high level of competence for midwives, and our work is also related to the safety of mothers and babies”* (29). Besides, there were some shortcomings about the hospital management systems, such as paying too much attention to employee rank, ignoring NQMs’ opinions and feelings, and focusing solely on number rather than quality of the work. *“Midwifery is a hierarchical system. It is based on midwifery-in-charge [and] also who has been here the longest or who has the most experience and it’s like you were in a food chain’”* (26). *“Midwifery practice requires me to actually give more loyalty to the hospital and do all the tasks that they expect of me in a day to save [them] from being sued or just to say, ‘These jobs have been done’”* (26).

## 5. Discussion

The systematic review of 14 qualitative studies was rigorously conducted by researchers trained in evidence-based nursing, contributing to a more in-depth and comprehensive understanding of experiences of NQMs during the transition period. The main findings indicated that NQMs faced challenges from multiple sources. These challenges mainly derived from the realistic clinical settings. They felt so stressed as a fresh midwife. At the same time, we also discovered their real needs and expectations. Therefore, to ease their physical and mental stress and further create a friendly work environment, transitional support for NQMs should be strengthened and the training system should be improved, which will play a positive role in reducing the resignation rate of new midwives.

Hospitals and schools should collaborate to facilitate a smooth transition to clinical practice for NQMs. Gap between theory and practice, high risk, high intensity, job insecurity are the main challenges NQMs faced during the transition. Complex interpersonal relationships and high-loaded work cause negative work experience, and affect adversely their physical and mental health (30). Therefore, it’s urgent to take various measures to help them meet challenges, and enhance their positive career experience. Hospitals and schools need deep cooperation to provide targeted career guidance to students. During the school, midwifery specialists can introduce the nature, significance, professional content and history of the midwifery profession in China and abroad, and share their professional experiences. Besides, the clinical practice is equally important. The midwifery training room should be available for students so that they can have a preliminary understanding and experience of the clinical midwifery work. This may help them identify and internalize their professionalism. Educators should pay attention to the combination of theoretical and practical teaching, cultivate students’ practical ability, so that they can better adapt to clinical after graduation. At the same time, attention should be paid to improve students’ psychological quality and improve their ability to cope with occupational stress.

The clinical transition support system should be improved to promote positive career experience for NQMs. The multifaceted, multi-disciplinary clinical support system has positive implications for the smooth transition of NQMs to new roles. According to studies, the level of clinical support new midwives receive during the clinical transition greatly influences their clinical competence (31). According to Thunes (32) and Fenwick (33), obstetrics students attribute their clinical success to the practitioners they work together every day. On the one hand, improving the support system can reduce the clinical responsibility and pressure of NQMs and prevent them from intentionally narrowing the scope of their practice for fear of taking risks; on the other hand, it can promote NQMs to maximize the professional role and provide maternal-centered midwifery services, so as to further improve the quality of maternal and infant health care.

Strengthen continuing education to ensure the sustainability of the NQMs team. Many NQMs often feel a lack of expertise and competence when face with complex clinical problems, and continuing professional learning becomes the expectation of most of them. And their new level of responsibility inspires the importance of continuous professional education in order to continue to provide safe care for women. Continuing education programs will help healthcare providers improve their professional competence and adapt them to rapidly changing and new roles (34–36). Particularly, midwives are one of the important healthcare providers, and their continuing education can enhance midwives’ ability to improve maternal and child health status (37).

Standardize the management system and provide a broad career exhibition path for NQMs. They have high expectations for regulated management systems, especially they want independent professional systems and independent professional behavior. At present, in many countries, the midwifery major still belongs to the nursing major (38), and midwives do not have independent professional title evaluation and promotion sequence, and lack of the corresponding assessment, registration and promotion system (38), which forms certain obstacles to the echelon construction of midwifery talents and the development of professional characteristics. Improving the standard management system of midwifery professional education and midwife registration is conducive to enhancing the sense of responsibility and autonomy of NQMs, providing a richer career development path, so as to promote the development of midwifery profession and the retention of talent resources.

## 6. Conclusion

This qualitative systematic review expounds the experience and feelings of NQMs during the transition to practice. Studies have shown that NQMs face multifaceted challenges, which have negative effects on their physical and mental health. NQMs are at a critical time in their career development, and properly guiding their role change is a difficult but important task. From the perspective of obstetric educators and clinical managers, this study suggests that hospitals and schools collaborate on guidance and intervention to improve clinical transitional support systems, standardize management systems, and strengthen continuing education. And thus, it helps NQMs make a smooth transition to clinical practice, gain positive career experiences, and provide them with a broad career path. This can contribute to the building and sustainable development of the midwifery workforce and better serve people.

## 7. Limitations

Although a systematic search was conducted using appropriate search strategies, according to the eligible criteria, only qualitative research or mixed-method studies from which qualitative data could be extracted were included. Gray literature and dissertations were not searched; only articles published in indexed journals in either Chinese or English were included. The omittance may have caused information bias. The included studies were of high quality, but two-thirds of the literature omitted information about the researcher’s theoretical or cultural background, which could have an impact on the results.

## Data availability statement

The original contributions presented in the study are included in the article/Supplementary material, further inquiries can be directed to the corresponding author.

## Author contributions

JS and XL: conceptualization, methodology, formal analysis, writing of original draft, and writing – review and editing. YL: conceptualization, methodology, writing of original draft, and writing – review and editing. YL, JL, RZ, and HJ: conceptualization, methodology, formal analysis, and writing – review and editing. All authors contributed to the article and approved the submitted version.

## Funding

This work was supported by: (1) Technical standard project of Shanghai Municipal 2022 “Action Plan for Science and Technology Innovation” (22DZ2203800), (2) Shanghai Shenkang Hospital Development Center Management Research Project (2022SKMR-18) and (3) Shanghai Shenkang Hospital Development Center Technology Standardization Management and Promotion Project (SHDC22022227).

## Conflict of interest

The authors declare that the research was conducted in the absence of any commercial or financial relationships that could be construed as a potential conflict of interest.

## Publisher’s note

All claims expressed in this article are solely those of the authors and do not necessarily represent those of their affiliated organizations, or those of the publisher, the editors and the reviewers. Any product that may be evaluated in this article, or claim that may be made by its manufacturer, is not guaranteed or endorsed by the publisher.
