# Supplementary material for: Experiences of newly qualified midwives during their transition to practice: a systematic review of qualitative research
Source: Front Med (Lausanne). 2023 Aug 16;10:1242490. doi: 10.3389/fmed.2023.1242490 (PMC10467268; doi:10.3389/fmed.2023.1242490)
Supplement: Supplementary file 1 [file Table_1.DOCX]

***Supplementary Material***

Experiences of newly qualified midwives during their transition to practice: a systematic review of qualitative research

Jinjin Shi^1†^, Xuemei Li^1†^, Yongqi Li^2†^, Ying Liu^3^, Junying Li^3^, Rongli Zhang^1^, Hui Jiang^4^

^†^These authors contributed equally to this work and share first authorship.

***Correspondence:** Hui Jiang [jianghuitest@163.com](mailto:jianghuitest@163.com)

**Supplementary Table 1.**

**ENTREQ checklist (Enhancing transparency in reporting the synthesis of qualitative research）**

| **No. Item** | **Guide and description** | **Reported on Page** |
| --- | --- | --- |
| 1. Aim | This qualitative systematic review aimed to understand the experiences of NQMs during their transition to practice and explored factors that promote or inhibit the progress. It is hoped that the review will provide professional healthcare workers a new perspective on guidelines and strategies, with the aim of producing a training platform for NQMs to help them in their professional roles and foster a positive working experience. | P2-3 |
| 2. Synthesis methodology | The JBI meta-aggregation approach was used to synthesize the data. Meta-aggregation is grounded in the philosophical traditions of pragmatism and Husserlian transcendental phenomenology, which is the most transparent and widely accepted methodology of all available for qualitative study synthesis for constructing high-quality systematic reviews of qualitative research. This approach accurately and reliably presents the findings by the original authors instead of re-interpreting the studies. | P3 |
| 3. Approach to searching | The Preferred Reporting Items for Systematic Reviews and Meta-Analyses guideline was adopted in this review. A three-step approach was used to identify the studies: (a) an initial limited search via PubMed, (b) a systematic search of electronic databases, and (c) a manual search of journal references. | P3; Figure 1 |
| 4. Inclusion criteria | **Participant (P):** NQMs that started clinical work with less than three years experience.  **Interest of phenomena (I):** The experiences of NQMs during their transition from education into practice. The focus was on their stressors, demands, and expectations.  **Context (Co):** Included studies were those performed during the transition from education into practice.  **Study design (S):** Qualitative research and mixed-method studies from which firm results came, including but not limited to phenomenology, grounded theory, case studies, action research, ethnography, and feminist research. | P4 |
| 5. Data sources | We systematically searched 12 electronic databases, including eight English language databases: PubMed, Web of Science Core Collection (via ISI Web of Science), MEDLINE (via ISI Web of Science), Cochrane Library, LWW (via OVID), CINAHL Complete (via EBSCO), Scopus, and ScienceDirect; and four Chinese databases: China National Knowledge Infrastructure (CNKI), Wanfang Database (CECDB), VIP Database, and China Biomedical Database (CBM). | P3 |
| 6. Electronic Search strategy | A separate search strategy was designed and results were limited to journal articles written in English or Chinese, and published before 15 February 2023. The query included five groups of keywords and MeSH terms combined with Boolean operators: (1) (new graduate midwives) OR (newly graduated midwives) OR (newly qualified midwives) OR (newly qualified nurse midwives) OR (new nurse midwives) OR (new midwives) OR (graduate midwives); (2) (transition) OR (transition period) OR (transition to practice) OR (transition programs) OR (change) OR (culture shock) OR (orientation) OR (standardized training) OR (pre-service training) OR (residency programs) OR (induction program); (3) (perception) OR (feel*) OR (experience*); (4) (qualitative research) OR (qualitative method) OR (qualitative study). Finally, the references of each qualifying paper were searched manually to identify further studies. The sample search strategy for PubMed is presented in Figure 1. | P5; Figure 1 |
| 7. Study screening methods | By comparing the evaluation criteria of qualitative research, two researchers (JS, XL) who had undergone qualitative research studies and training in evidence-based methods were selected to conduct the study. The two researchers used the “JBI Evidence-Based Quality Evaluation Criteria for Qualitative Studies in Evidence-Based Health Care Centers” for the final independent evaluation. Each was evaluated by “yes”/ “no”/ “unclear”/ “not applicable” – and if all 10 items were “yes”, the possibility of bias is minimal and is in category A. If partially met, the possibility of bias is category B. If all items are “No”, the possibility of bias is considered high and is in category C. After independent evaluation, the results of the two individuals were compared and a third party re-evaluation was in place in case of disagreement. Finally, the literature with a quality level of C was excluded. | P4; Figure 2 |
| 8. Study characteristics | The 14 studies were conducted in the following countries: China (n = 1), Malawi (n = 1), South Africa (n = 2), the Netherlands (n = 2), Ireland (n = 2), the United Kingdom (n = 3), and Australia (n = 3). These studies involved 238 NQMs. Study designs included qualitative action-research approach (n = 1), phenomenological approach (n = 4), qualitative descriptive approach (n = 5), mix-method study (n = 2), ethnography (n = 1), and one study described as qualitative without a specific approach (n = 1). All the studies were published after 2008 and were original articles. Study characteristics are presented in **Table 2**. | P5; Table 2 |
| 9. Study selection results | A total of 509 relevant papers were initially searched from the database. After removing duplicates, 453 were collected through NoteExpress. The two researchers independently read the titles, abstracts and keywords to obtain 20 papers, after reading the full text, 14 papers were included. The detailed search and screening process is shown in **Figure 2**. | P5; Figure 2 |
| 10. Rationale for appraisal | The two researchers used the “JBI Evidence-Based Quality Evaluation Criteria for Qualitative Studies in Evidence-Based Health Care Centers” for the final independent evaluation. Each was evaluated by “yes”/ “no”/ “unclear”/ “not applicable” – and if all 10 items were “yes”, the possibility of bias is minimal and is in category A. If partially met, the possibility of bias is category B. If all items are “No”, the possibility of bias is considered high and is in category C. After independent evaluation, the results of the two individuals were compared and a third party re-evaluation was in place in case of disagreement. Finally, the literature with a quality level of C was excluded. | P4 |
| 11. Appraisal items | JBI Qualitative Assessment and Review Instrument. | P4-5; Table 1 |
| 1. Appraisal process | Appraisal was conducted independently by two trained independent reviewers (JS, XL). When the evaluation results conflicted, the third researcher (YL) decided. | P4 |
| 13. Appraisal results | The quality of the included literature was evaluated and the results were all B grade or higher. The assessment of study bias are presented in **Table 1**. | P5; Table 1 |
| 14. Data extraction | According to the JBI meta-aggregation, qualitative data were extracted in two steps. First, publication details (author’s name, publication year, country or region, research aim, research design, method of data collection, sampling and data analysis, participants) and the findings were extracted. Second, statements about the experiences of NQMs during their transition to practice were extracted for a subsequent meta-synthesis across all included studies. The two reviewers (JS, XL) independently evaluated the plausibility of each finding and identified them into three levels:  (1) Unequivocal (U): relates to evidence beyond a reasonable doubt, which may include findings that are matter of fact, directly reported/observed, and not open to challenge.  (2) Equivocal(E): those that are, albeit interpretations, plausible in light of data and the theoretical framework. They can be logically inferred from the data.  (3) Not Supported (NS): when 1 nor 2 apply and when most notable findings are not supported by the data. The extracted findings that had similar meanings were aggregated to form new categories. Eventually, these categories were further synthesized to generate more comprehensive findings, called “synthesis findings”. | P4-5; Supplementary Table 2 |
| 15. Software | NoteExpress software was used. | P5 |
| 16. Number of reviewers | 3 reviewers (JS, XL, YL) | Three reviews |
| 17. Coding | JBI meta-aggregation did not use the technique of coding. | NA |
| 18. Study comparison | The extracted findings that had similar meanings were aggregated to form new categories. Eventually, these categories were further synthesized to generate more comprehensive findings, called synthesis findings. | P4 |
| 19. Derivation of themes | The process of deriving the themes or constructs was inductive. Only unequivocal and equivocal findings were included. Findings that possessed similarity in meaning or addressed a similar phenomenon together were summarize to form new categories. Ultimately, these categories were subjected to further synthesis to generate more comprehensive findings, known as synthesized findings. | P5; Supplementary Table 3 |
| 20. Quotations | Supplementary Table 2 provided findings and quotations from the primary studies to illustrate themes and constructs, and identify whether the quotations were participant quotations of the author’s interpretation. | Supplementary Table 2 |
| 21.Synthesis output | The researcher extracted 84 findings from the 14 papers and summarized them into eight categories. From these categories, three synthesized findings emerged: multi-dimensional challenges, physical and emotional responses, and demands and expectations. The detailed process of synthesis is reported in **Supplementary Table 3**. | P5; Supplementary Table 3 |

**Supplementary Table 2.**

**Findings extracted from the included studies with illustration**

| **Wier, J et al. (2022)** | |
| --- | --- |
| Finding 1 | Expectations of self (Unequivocal) |
| Illustration | “You expect yourself to know everything now you’re newly qualified…and it takes a while to realize that you don’t…” (June, NQM, P4)  “You want to be the best of the best…I probably put too much pressure on myself…I just need to have confidence and take a deep breath….and I’ll be alright…but then every once I have a little panic…” （Belinda, Yr. 3 SM，P4） |
| Finding 2 | Expectations of others (Unequivocal) |
| Illustration | “And then suddenly you’re qualified, and I am worried about the expectations the midwives have for us as newly qualified [midwives]…” (Belinda, Yr. 3 SM, P4).  “I think are they going to perceive me as: well, you’re newly qualified and you need to be able to do this, and I think I shouldn’t have to take on the mantle of their expectations and it’s about how I say: actually, that’s your expectation, not mine…” (Donna, Yr. 3 SM, P4).  “As a student you have a security blanket and allowances are made…you can ask for help and support…if I don’t know what to do I’m just going to say……if their expectations of me are higher, so be it…it wouldn’t be safe practice not to ask for help…whatever their expectations are …” (Elizabeth, Yr. 3 SM, P4).  “The idea of working where you’re known as a student…I feel like there might be more of a level of expectation [with staff indicating] …” You know how it works here, go and do …” (Debbie, Yr. 3 SM, P4). |
| Finding 3 | Accessible support (Unequivocal) |
| Illustration | “The support I’ve had has been amazing…I couldn’t, I can’t fault it….” (Claire, NQM, P4)  “The majority of the midwives have been amazing…with just a couple that I don’t feel comfortable going to…they can be a bit intimidating, so I steer clear and just get on with my job…” (Cathy, NQM, P4)  “I think that the biggest worry between being a student and being newly qualified is that you haven’t got that safety net anymore… you’re still newly qualified and going out on your own making your own decisions but you still need that sup- port…” (Chloe, Yr. 3 SM, P4)  “Having someone approachable and accessible that I could go to and speak to for support is really important…” (Becky Yr. 3 SM, P5)  “It feels like walking in the dark [not knowing what’s going to happen] …and you hope you’re going to have support” (Chloe, Yr. 3 SM, P5).  “My relationship with my personal tutor…gives me confidence to talk to somebody about problems…I hope I can continue with that…and I find someone that I can have that confidence in…” (Ellie, Yr. 3 SM, P5).  “It’s really important to have someone to support us, that we can go to for emotional support more than anything…” (Debbie, Yr. 3 SM, P5). |
| Finding 4 | Peer support (Unequivocal) |
| Illustration | “You could have a band 5 forum…just to sit and chat…and talk together about how we’re feeling would help…” (Lilly, NQM, P5).  “[having peer support] should be part of the support process…. an opportunity for us to feel like our concerns are being listened too…It’s not just us talking amongst ourselves…” (Fay Yr. 3 SM, P5).  “We’re really good as a support network for each other… and losing that is one of the anxieties I have because you don’t know who you might end up working with……Its building that new safety net of friends and colleagues again…” (Becky, Yr. 3 SM, P5).  “We have our own little online group… to support each other…I hope we continue this…” (Donna Yr3. SM, P5) |
| **Simane-Netshisaulu, KG (2022)** | |
| Finding 5 | Labor ward viewed as a negative working environment (Unequivocal) |
| Illustration | “The delivery room is so hectic, at times one may even think of removing one’s shoes and walk barefooted. Oh! I am tired of working in the delivery room.” (Participant 1, 24 years old, female, P4) |
| Finding 6 | Supervisory and teaching roles and responsibilities of newly qualified midwives (Unequivocal) |
| Illustration | “I am glad that I successfully made it to be a professional nurse, the problem is the fact that all junior staff members expect that I provide answers to their problems. When I think about that I feel like not coming on duty. Mhhhhhh... it’s so awkward to be expected to take a supervisory and teaching role.” (Participant12, 23 years old, male, P4)  “One thing that makes me scared is the reality that I must stand as a professional nurse and supervise students as well as patient care. We were never given a chance to decide on patient care during training, our responsibility as students was to carry out instructions from the doctors and professional nurses, but now you are expected to make decisions that affect the patients’ lives. It’s not easy, it’s stressful.” (Participant8, 22 years old, female, P4) |
| Finding 7 | Administrative roles of newly qualified midwives (Unequivocal) |
| Illustration | “When we were students, we were never given any chance to practice managing the unit, but suddenly you are expected to manage the unit including patients, staff members, equipment and supplies. This is not easy. Especially because you don’t feel confident enough to delegate duties to some members of staff.” (Participant9, 25 years old, male, P4) |
| Finding 8 | Negative attitudes of experienced midwives (Unequivocal) |
| Illustration | “The relationship between us and some senior members is ok, however, some are unfriendly. The unfriendly ones give bad and demotivating remarks when we seek for assistance. It’s bad.” (Participant11, 24 years old, female, P4)  “What can you do? Because if you seek for assistance, they say you are testing them, if you keep quiet they say you consider yourself as a better person who knows much. What can you do to make them happy?” (Participant17, 25 years old, male, P4) |
| **Mtegha, Mathews Brave et al. (2022)** | |
| Finding 9 | Theory practice gap (Unequivocal) |
| Illustration | “Upon reaching the ward, I found that most of the guidelines like HIV guidelines, and some reproductive health standards had changed. There were also new things like CPAP (continuous positive airway pressure). So it was really tough for me as I was referring to old things, yet, the practice had changed on the ground” (NMT-F- Participant4, P6).  “It was kind of difficult for me to fit in because when I was trying to do what I knew from training, people were always against me. They could say, we don’t follow theory rather hospital policies and management guidelines…What is learnt in theory is not always what is on the ground. It is very different” (RNM-F-Participant1, P6)  “I wanted to manage my clients online with what I learnt in class, only to realize that in practice most conditions are managed differently. The situation made me feel lost in wilderness” (NMT-F-Participant2, P6)  “At first, since I was just coming from college, I had more knowledge compared to practical skills. It took me time to adapt and acquire the necessary skills to match my level of theoretical knowledge acquired during training” (RNM-M-Participant3, P6). |
| Finding 10 | Lack of confidence and skills (Unequivocal) |
| Illustration | “I was excited when I got a job. But seriously, coming to the ward, I felt not prepared to provide care unsupervised. I even told them that I was not comfort-able to do anything in nursery without support from colleagues...and for the first 2 weeks, I was calling experienced staff or colleagues to be there with me whenever I was doing something. I wanted to at least be guided when doing the procedure until I gain the skills” (NMT-F-Participant2, P6). |
| Finding 11 | Inadequate resources (Unequivocal) |
| Illustration | “….. human resource is a challenge…Despite the nursery ward being one of the busy wards, there are times that you are alone on duty and you are expected to do all the activities…if you have pre-mature babies, you need to resuscitate them, you have to administer medication, you have to monitor vital signs… it’s not easy. You cannot even have time to rest. But the sad thing is that seniors do not understand this” (NMT-F-Participant2, P7)  “Sometimes I could work here alone in postnatal ward with 20 to 24 women. Honestly, I was not enjoying the work because it was too much for me” (RNM-F-Participant4, P7)  “The transition process itself is stressing, shortage of resources magnifies the stress. You know, there are times when you fail to perform a procedure because of lack of resources. I remember we once lost a mother to postpartum hemorrhage. It is not that we did not know what to do to save her, but we did not have resources like normal saline…You know psychologically and as a new graduate, you become affected and obviously you cannot enjoy the profession… the issue of resources was a very big challenge for me” (NMT-M-Participant3, P7). |
| Finding 12 | Lack of transitioning support system (Unequivocal) |
| Illustration | “We were not under any program. The first week I was just working with anyone who was on duty for support. Later, I could follow or consult peers and seniors whom I thought were competent in their midwifery skills (NMT-F1-Participant5, P7).  “Transition is always difficult without support. It is like you are coming from a place where you were used to and you are going to a new place where you do not really know what happens there…so in such situations mentorship is needed” (RNM-F-Participant1, P7).  “The challenge we have in Malawi is the long waiting time for newly qualified nurse midwives to be employed. By the time you are employed you have forgotten all that you learnt at school. I waited for a year for employment. When I got the job, I felt like someone who has not been trained in midwifery because it was difficult to recall the management of conditions.” (NMT-F-Participant2, P7)  “It took a long time from graduation to when I started practicing as a midwife. I almost forgot everything. But support from experienced mid-wives helped me to get on track. By and by I started remembering the management of most conditions.” (NMT-F- Participant4, P8). |
| Finding 13 | Workplace conflict (Unequivocal) |
| Illustration | “Some nurses could say, you have a degree and you know all these things. Why are you asking me? Next time you can’t ask that person again for guidance and definitely you won’t learn how that skill is per-formed” (RNM-F-Participant1, P8).  “I had to find some midwives who were not giving room to teach or mentor us. They could say, you have just graduated from school …we thought that qualifies you are knowledgeable and skilled?” (RNM-M-Participant5, P8) |
| **Cazzini, H et al. (2022)** | |
| Finding 14 | Feeling challenged (Unequivocal) |
| Illustration | “I think the first word that comes to my head is overwhelming.” (Pippa, P2)  “You wanted someone to double-check everything you were doing even though you knew you could do it...I found that I questioned myself a lot more than when I was a student…because you knew it was your registration now.” (Roisin, P3) |
| Finding 15 | Set high standards for yourself (Equivocal) |
| Illustration | “I worked as a nurse earlier on but it’s completely different because you’re more independent as a midwife because you have to make more choices you have to have more clinical judgment…more pressure, more responsibility and being more accountable for what I do.” (Avril, P3)  “You have to be on the ball…there is no time to learn on your own pace you just have to know everything...that adrenaline rush and you just need to know everything.” (Emily, P3) |
| Finding 16 | Attention and evaluation from colleagues (Unequivocal) |
| Illustration | “I think they judge you if you don’t get it right the first time...sometimes you’re afraid to ask because you’re a midwife now.” (Avril, P3)  “Everything is a test, everyone is “all eyes on you… you feel like, if I get one thing wrong…I’m the snake in the nest.” (Pippa, P3) |
| Finding 17 | Too busy (Unequivocal) |
| Illustration | “Our workload is so much that my main priority in the day is managing my workload…there’s too much work and not enough support”. (Laura,P3)  “When there is a staff shortage, the junior staff are always moved around a lot…I feel we are just like a number”. (Emily, P3) |
| Finding 18 | Disappointment (Unequivocal) |
| Illustration | “For me, being with the woman is just a part of my soul…but I don’t get a lot of time to do that…I have to do a lot of things rather than actually being with woman…I suppose the environment that we work in…doesn’t really value…the real side of the care that we give...you’re just permanently in survival mode.” (Laura, P4) |
| Finding 19 | Getting stuck in learn from mistakes gaining confidence (Unequivocal) |
| Illustration | “We learn in college but really you learn the most by actually doing it.” (Noelle, P4)  “It is through trial and error that you learn…just to put myself in there and just learn how to swim.” (Emily, P4)  “There’s no tool or anything that you can put in place that’s going to replace the experience that you get from just being in there and doing what you need to do and seeing how the team operates and how the wards operate.” (Pippa, P4) |
| Finding 20 | Transitioning to practice in a familiar environment eased their transition. (Unequivocal) |
| Illustration | “You see, I’m lucky in a way that I trained here, so I know where everything is and how everything Works”. (Noelle, P4)  “Well as I trained here...I think I know the hospital and I know how everything works”. (Avril, P4)  “I had clinical skills support for a week or two. And that was good for the more practical side to Things”. (Noelle, P4) |
| Finding 21 | The need for support from an approachable colleague in the clinical area (Unequivocal) |
| Illustration | “It probably would have been nice at the beginning to have maybe a linked midwife, kind of like a buddy type of system. I think that probably would be nice just someone that you could ask questions to”. (Noelle, P4)  “The manager on the ward was excellent, she was always checking in with you making sure that you were doing okay”. (Roisin, P4)  “We have a really good manager/leader, she’s good clinically she can meet us emotionally and she was just…approachable”. (Laura, P4)  “If you got a really good, friendly, supportive person, that day you felt great”. (Noelle, P4) |
| Finding 22 | Additional training needs (Equivocal) |
| Illustration | “[We need] more training, maybe high dependency…practical...especially because we are direct entry, I mean you kind of know “diddly squat” about gynae cases.” (Sheila, P5)  “I think the direct entry students might benefit with just basic skills like even cannulation and all those things”. (Emily, P5) |
| Finding 23 | Team-building events would be beneficial (Equivocal) |
| Illustration | “[Following an emergency], your manager would debrief you on that [situation] - she may say “well done” “this is what you can do the next time” or “you need to work on this or that”. (Noelle, P5)  “Some kind of protected time...to be able to reflect with my colleagues would be incredible”. (Laura, P5)  “We don’t really do anything together ever as a group, so that’s why it probably takes a bit longer to feel like you are part of the team”. (Sheila, P5) |
| **Simane-Netshisaulu, K et al. (2022)** | |
| Finding 24 | Newly qualified midwives expressed dissatisfaction about the mentoring process. (Unequivocal) |
| Illustration | “Some experienced midwives observe you as they pass, if you are doing something right they just keep quiet. If what you are doing is wrong, some will just say ‘do this and this’. Some say ‘ask your colleagues to help you. This is bad because as a new graduate, I need somebody who will take me by my hand and show me the way as my mentor.” (P4)  “Honestly speaking there is no supervision and mentoring. When a patient is in labor, I progress her and even deliver her alone without any supervision or assistance. It is really unfair.” (P4) |
| Finding 25 | Graduates described midwifery units as being non-conducive for effective transition. (Unequivocal) |
| Illustration | “It is not that we were not taught during training….no, we were taught, and we know how to do these procedures. But you need somebody who is experienced to support you as you stand so that you gain confidence to perform procedures as an independent practitioner.” (P5)  “The situation is not good at all; in some instances, you have to learn through trial and error. I was so scared of resuscitating a new-born baby, until one day in which I had to practice it all by myself. Fortunately, the baby cried whilst I was still struggling with the tubing. We were well prepared academically, but you need to have more time in scary areas such as resuscitation of a new-born baby.” (P5) |
| **Donovan,Helen et al. (2021)** | |
| Finding 26 | Physical exhaustion (Unequivocal) |
| Illustration | “It’s just exhausting, just physically. Some days you just need to sleep” (P2NM, P4).  “Physically very draining. Especially if it’s understaffed and you end up doing a double shift because someone needs to do it” (P10NM, P4).  “If you do three twelve-hour shifts in a row, and you have three days (off), one or two days of that is recovery. It’s not as if you’ve got those days off, you’re recoverin.” (P6N, P4).  “There’s no regularity to it, almost every day it’s a different time you’re waking up and going to bed” (P22NM, P4). |
| Finding 27 | Emotional exhaustion (Unequivocal) |
| Illustration | “I was absolutely terrified just because I hadn’t done it (nursing) for so long . . . and I would be like, I don’t know if I can do this. I don’t know what I’m doing” (P7NM, P4).  “Just the loneliness was probably the most emotionally draining thing.” (P15NM, P4) “It’s really important that you are able to debrief with friends and family because you will say things to friends and family that you wouldn’t say to work colleagues.” (P16N, P4)  “So I did spend a lot of my time on the phone with friends and family. They were really supportive.” (P7NM, P4) |
| Finding 28 | Mental exhaustion (Unequivocal) |
| Illustration | “You’re learning so much . . . there’s not a shift where you don’t learn something . . . your brain doesn’t stop the whole time” (P22NM, P4).  “I find that even on my days off I’m going to bed at eleven at night because my brain isn’t actually disengaging” (P9M, P4). |
| Finding 29 | Sleep deficiency (Unequivocal) |
| Illustration | “The first couple of times I did a 12 hour shift I thought I was going to fall asleep on my feet.” (P19NM, P5)  “I would sleep [as soon as I got home] and I would wake up for the next shift the following morning. So, I needed 12 or 13 hours of sleep everyday just to survive.” (P12NM, P5)  “I don’t think I will ever get used to shift work! It’s almost debilitating… you just start to doubt yourself and I think ‘Am I safe practicing when I’m this tired or this exhausted?” (P1NM, P5).  “You end up calling in sick because you realize that you’re not safe to practice when you’re that tired” (P21NM, P5). |
| Finding 30 | Work–Life Balance(Unequivocal) |
| Illustration | “Around here I spend a lot more of my time at work. I don’t know how to balance it” (P13NM, P5)  “There is no work–life balance. It is all work and no life” (P17NM, P5).  “I hadn’t seen mum and dad in ages, so I felt like I was missing out on the good parts of my life through working” (P2NM, P5).  “Our rosters are out 2 and 3 months in advance, so I have learnt to plan things ahead of time if I wanted to have any sort of a social life” (P8NM, P5). |
| Finding 31 | No time outside of work (Unequivocal) |
| Illustration | “If I had more time, to read up on these areas . . . It gives you time to look things up you don’t know . . . to reflect and recover” (P22NM, P5).  “I was crying all the time and had to take a week off” (P9M, P5). |
| **Kool, L E et al. (2020)** | |
| Finding 32 | High workload (Unequivocal) |
| Illustration | “The difference [with primary midwifery care] is that you have a whole maternity ward, we have seven delivery rooms which we have to take care of and they are sometimes really full with five [patients] and when a referred patient comes in and then you have to do your consultation. You have to be really good in keeping an overview and setting your priorities!” (Participant7, P4) |
| Finding 33 | Becoming a team member (Unequivocal) |
| Illustration | “There are a lot of dynamics in the hospital …And it took me some time to realize which disciplines are involved and which agreements are made per hospital, and about protocols. And even if you have a protocol, the usual way of doing things can be different, and it takes a while before you know this. It differs per hospital, but also per obstetrician it is different again, and per nurse and per primary care midwife. That is so diverse, it really took me a while before I really knew how it works and I still run into issues now and then.” (Participant3, P4). |
| Finding 34 | Additional midwifery skills and procedures (Unequivocal) |
| Illustration | “Especially with the CTG [cardio toco gram], you are immediately thrown in at the deep end. You have to work in practice with the CTG and it remains difficult and partially subjective. What one per- son thinks can be different from another and you must have a lot of experience with it if you want to be able to take advantage of it. And then you sometimes make choices that you think would not have been necessary. So that in particular - and even more so at night when you are alone in the delivery room.” (Participant3, P4) |
| Finding 35 | Providing care for women (Unequivocal) |
| Illustration | “…about the ethical things that I sometimes find difficult. We have a large refugee center nearby and we sometimes have difficulties with people from a different culture who want different things. Partners with different opinions about pregnancy and birth. Once I felt enormously threatened by a partner, because I did not work like the midwives act [in their country of origin]. And those are tricky thing… We really learned it differently from how they want it. Yes, and then you do want to go a bit with the culture, but yes, you naturally also want to continue to do medically responsible things.” (Participant4, P4) |
| Finding 36 | Job insecurity (Unequivocal) |
| Illustration | “I now increasingly have the end date in my mind… I’m still looking at other job vacancies. Yes, because they cannot give me clarity yet. That feels … quite annoying because I really feel like I am a part of this team ... and then you are confronted with the fact that you do not have a permanent contract yet.” (Participant2, P4) |
| Finding 37 | Working in a team (Unequivocal) |
| Illustration | “Teamwork is really important, that actually determines every- thing in your work, I think, because you need each other.” (Participant17, P4).  “... my team manager was actually the one who was responsible for the focus on my settling-in period support. I had an evaluation interview with her on a weekly basis, just very briefly, to see where are you, how are you doing and how do we continue?” (Participant10, P5) |
| Finding 38 | Working with women (Unequivocal) |
| Illustration | “If it is just a beautiful birth: mother and child are doing well. Or, if people when they leave say: ‘oh, thank you’. Or a follow up check where people are just satisfied with you. That’s the best thing. Or sometimes also a heavy situation that is nicely solved, a shoulder dystocia that you get out, that ends well.” (Participant14, P5) |
| Finding 39 | Variety of the work (Unequivocal) |
| Illustration | “An acute situation that ends well, that was very thrilling ... I like the challenge when someone has a hemorrhage post- partum. I think okay, what can I do in order for her to be fine. Yes, I really like that kind of action.” (Participant13, P5) |
| Finding 40 | Employment conditions (Unequivocal) |
| Illustration | “Stability in employment conditions such as a secured contract, was also experienced as a resource.” (P5)  “[A roster] It gives me much more comfort in secondary midwifery care knowing that at the end of your shift, you hand over the phone and not take it to bed with you.” (Participant3, P5) |
| Finding 41 | Personal resources (Unequivocal) |
| Illustration | “I also dared to make decisions and I dared to pick up [tasks] independently and it is really not that I needed help with any- thing and everything. I think that I can generally work independently.” (Participant13, P5) |
| Finding 42 | Personal demands (Unequivocal) |
| Illustration | “But feelings of uncertainty, can I do it, am I doing it right? And sometimes sad feelings, I’ll never get the hang of it. For ex- ample, if I had to start an induced labor, and then it didn’t work as I expected… And then my colleague told me: we can easily break the membranes. And then I was so embarrassed. And well then, I started to break the membranes and then I didn’t succeed. And then I let her do it. Then just disappointment, gloom, insecurity…” (Participant10, P5)  “I am sometimes so much in doubt. Is it perfectionism, or is it some form of being afraid to fail. I have noticed more than ever since my graduation that you carry responsibility for mother and child, that is a certain pressure that you feel. And then you think that can indeed be fatal… And perhaps it is a factor that I can be sensitive or afraid of doing things wrong.” (Participant 2, P5) |
| **Kool, L et al. (2019)** | |
| Finding 43 | Working as locum (Equivocal) |
| Illustration | “Yes, you know …you have no job-security, so you take all the work you can get everywhere. And, yes, I can recall, the insecurity that belongs to locum midwifery … that increases pressure”. (A1) You want to work everywhere and therefore you will cross personal boundaries.” (D5, P4) |
| Finding 44 | Balancing work private life (Unequivocal) |
| Illustration | “When I was working, everything went all right, but when I was at home, I collapsed so to speak. Then, emotions came up, so to speak.” (B1, P4) |
| Finding 45 | Colleagues were mentioned as both a demand and a resource. (Unequivocal) |
| Illustration | “I can deal very well with my colleagues, but sometimes when I am in doubt about a small issue, I think: yes, I can call my col- league, but then they could think: why is this employee working for us?” (D7, P4)  “It’s just the simple approach, you may call me in the middle of the night. Having a colleague available…just to deliberate with them. That idea gives me confidence, you know.” (B6, P5) |
| Finding 46 | Working in different regions was mentally demanding for NQMs. (Equivocal) |
| Illustration | “…to start an emotional conversation. Because, during placements…as supervising midwife, you do not leave that kind of responsibilities to students.... So, as a student I did not practice such difficult conversations.” (B4, P4) |
| Finding 47 | Working autonomously was identified by participants as both a job demand and a job resource. (Unequivocal) |
| Illustration | “When you are facing a home delivery and eh, the maternity care assistant is nine out of ten times too late. Then you really stand alone.” (A5, P4) |
| Finding 48 | Organizational tasks (Unequivocal) |
| Illustration | “.. I took a lot of time at home, preparing myself for the prenatal visits. Afterwards, I went to the practice and still ran out of time. Therefore there was a lot of catching up to do.” (A4, P4) |
| Finding 49 | “Contacts with clients” as particularly motivating. (Unequivocal) |
| Illustration | “When I support a woman in labor…. that is why I chose this profession. Then it is easy to get out of my bed in the night. Moreover, I feel that my work is my passion, and my passion is my work.” (A3, P5) |
| Finding 50 | Personal resources (Unequivocal) |
| Illustration | “Setting boundaries on both levels: management tasks and providing care. A good midwife does not have to work seven days a week!” (C3, P5) |
| Finding 51 | Personal demands (Unequivocal) |
| Illustration | “That you wrote down a huge amount of words in a detailed text about what had happened. That other people know what you did during your shift and I always prepared myself for the upcoming shift. And before I started a consultation with a client, I read all the reports about this client… That costed me a large amount of energy; I was exhausted.” (C4, P5).  “I was educated abroad. So I felt I had to prove myself, you know... it took a while before I had a job as a midwife in the Netherlands. And, after a while, you become insecure…I had a strong feeling that I have to prove myself.” (A2,P5)  “My pitfall is that I cannot let go of the thoughts about the things I did not do right. That I continue to worry.” (C1, P5) |
| **Norris, S (2019)** | |
| Finding52 | Fell traumatized and angry (Unequivocal) |
| Illustration | “If I’m completely honest, my feelings from when I first started are just completely vulnerable and on my own and really unsupported on the ward. In fact, I’ve really struggled to kind of have any issues or anything I’ve had to be recognized, and any note taken of how difficult it is.” (FG1.1, P3)  “You need to know how to look after yourself because no one else looks after you. It’s hard, I mean they talk about it but the actual doing after that, well how do I? It builds resentment. It’s really difficult.” (FG1.3, P3)  “I find that I’m exhausted and tired nearly all the time especially when I have both day and night shifts in the same week… I have tried to make sure I have plenty of healthy food in the house and try to go out. However, I still sleep uneasily before a day shift, I have to solve that one.” (P010, P3) |
| Finding53 | Uncertainty and considerable challenge (Unequivocal) |
| Illustration | “These girls have never set foot on that ward until their first shift. They would come to me and say: ‘I’ve been crying in the toilet today.’ If some of them were here now, they’d be saying to you: ‘I cried for probably most shifts for the first two or three months.’ They coped so much worse and they didn’t know the people, they hadn’t met the Band Sevens upstairs, they didn’t know their names, such a small thing. They didn’t know who they could ask. They didn’t know the midwives who would be helpful to them. They just saw them all as navy. It was scary, a lot of them struggled.” (FG1.1, P4) |
| Finding54 | Cope was admired by colleagues and asking for help was a weakness. (Unequivocal) |
| Illustration | “You don’t want to be seen as the one who’s not coping, you don’t want to be the one that, after you’ve had handover and they’re like, ‘Oh fine, don’t worry.’ Then they go to the desk and they’re like: ‘I don’t want to come on to work after her, she leaves everything for the night staff.” (FG1.3, P4)  “It’s just having the guts to just keep asking and asking the right person, and asking somebody and not feeling a fool for asking, because sometimes they make you feel a fool in front of the women you’re looking after.” (FG1.1, P4) |
| Finding55 | Relationships with colleagues (Unequivocal) |
| Illustration | “Most of the time the midwives were as supportive as they could be. Most of them were really nice… they were under the same pressure so they did as much as they could.” (FG1.3, P4)  “The Band Seven that was on was fab. We actually went to theatre for it all and needed the crash team and, once everything had settled, she called me out and put another midwife in and said: ‘Go and have a biscuit and a cup of tea, sort yourself out and then you go back and do your notes.’ I think sometimes that’s what you need – someone to look after you.” (FG2.2, P5) |
| Finding56 | Relationships with women (Unequivocal) |
| Illustration | “I had a shift and one of the women was really upset about not going home; she was crying. I just hadn’t been able to get to her because I had a social worker case as well. I’d spent three hours on the phone to the social worker. Seven discharges.” (F1.3, P5)  “In my break I was feeling ill and she was saying: ‘I’m going to make a complaint about you, you didn’t tell me I was going to be here until seven in the evening.’ It’s a communication thing.” (F1.4)  “I found that I didn’t want to say I was an NQM because then…I think there’s an air of do they really want you looking after them? I would say I was new with the Trust.” (FG1.2, P5) |
| Finding57 | A new beginning (Unequivocal) |
| Illustration | “I’m naturally quite a quiet person, not shy, but just quiet. I have to learn to portray a boldness of character and also that it is a process of learning to have confidence in my own practice. Confidence in what I know and confidence in what I don’t know.” (P010, P5) |
| **HUANG Shu-rong et al. (2017)** | |
| Finding 58 | High risk, high intensity, high stress (Unequivocal) |
| Illustration | “The workload is heavy, and I feel busy and tired a lot of the time, especially at night...I feel like I can't make up for the energy I've expended after sleeping for days. Secondly, psychological pressure, always have to worry about too many things, the child did not come out before to care about fetal heart is good, amniotic fluid is good, labor progress ah......The child came out to care about the child score is not good, how the mother's contraction, there is no bleeding, there is no timely solution to urinate, wound long...”（Participant9,P2）  “There are often a lot of emergencies in the delivery room, the seemingly familiar natural birth often hidden a lot of unusual dangers. So remind yourself to be careful and to work hard.”（Participant3,P2）  “Especially under the guidance of the national policy to open the second child, the midwifery profession has faced great challenges, a wave of scarred uterus, high blood pressure, diabetes, elderly women.” （Participant14,P2） |
| Finding59 | Full of joy of rebirth, a sense of accomplishment and value (Unequivocal) |
| Illustration | “Midwifery is a sacred profession. When the baby is born, the midwife is the first person in the world to embrace the baby...It will make me feel the joy of work, without the oppressive working environment of oncology department.” （Participant4,P2）  “See the mother in peace, happy family, feel the whole world is full of sunshine.” (Participant1,P2）  “I feel it is a great and happy thing to welcome a new life...Sharing the joy of each family is also a relief from hard work.” （Participant9,P2）  “Like the delivery room, I think it is a skill, with a stronger sense of value and a higher sense of achievement...I was attracted to welcome new life every day. I chose to study medicine because I wanted to choose a job that can help others. I thought that I would not be successful in my long life, but at least I should try my best to do something in a small area.” （Participant3,P3）  “When you hold the baby to family members, they will cry with joy, and you will be touched, and you will feel that what you have done is worth it.” (Participant9,P3） |
| Finding60 | There is a gap between the actual work and the ideal. (Unequivocal) |
| Illustration | “The work is urgent and busy, many times the labor room manpower and equipment is not enough, easy to make midwifery work into assembly line operation, so that the theory of gentle midwifery model is missing, midwifery staff lack of love and patience, easy to lead to the ideal and reality of the gap and sense of loss.” (Participant7,P3）  “Long working hours, high work intensity... Can’t realistically give pregnant women professional midwifery technical support, can’t play the initiative of midwives, just deliver, deliver.” (Participant4, P3） |
| Finding61 | Look forward to diversified career development (Unequivocal) |
| Illustration | “As a young midwife, I am well aware of my lack of ability and experience, so my recent plan is to study hard, reflect more, summarize more, and improve myself...Then do something more, such as open a public account to write some popular science articles, use technical skills, or enroll in graduate school to further improve myself.” (Participant2, P3)  “Do some research on midwifery, do some topics...There is relatively little research on midwifery.” (Participant8, P3)  “Strengthen the study of professional English and spoken English, and have the opportunity to learn advanced ideas abroad.” (Participant12, P3)  “I hope to continue my study in midwifery and continuously improve my skills in technical operation and clinical thinking. I hope I can assist Cory to carry out midwife clinic in the future.” (Participant13, P3) |
| Finding62 | See the development potential of the profession and desire to standardize the industry management. (Unequivocal) |
| Illustration | “The efforts of the predecessors to appeal and voice, innovation and reform, for the development of the midwifery profession continue to open up a way, generally speaking, the midwifery profession development prospects are relatively bright.” (Participant7, P3)  “First of all, the work of midwives and nurses is very different, but still attached to the nursing, midwifery had better be independent from nursing, forming their own standardized set of management system.” (Participant3, P3)  “For young midwives, there should be a standardized training system, and they should have standardized training just like clinicians. After all, this line of work requires a high level of competence for midwives, and our work is also related to the safety of mothers and babies.” (Participant4, P3) |
| **Hobbs, J A et al. (2012)** | |
| Finding63 | Old school midwives (entrenched viewpoint) (Unequivocal) |
| Illustration | “[Name] is always like this. She is an old school midwife and so particular. You have to adapt to this all the time or explain why you want to do things differently.” (Ann, P6)  “We DO things that we know are going to make it not NORMAL! Some of it is old school stuff actually. I have got this policy in my head. My policy is, if it is going normal then I am not interfering. I am not Arming someone for no reason, because the less I interfere the better, quite frankly. But I have had people, ‘Oh well I have always done it the minute they are arable, I ARM them’. ‘Why wouldn’t I?’ she said it like that. And that is a bit of an old school thing, isn’t it? And it is a bit like a conveyor belt sort of thing.” (Faye, P6)  “I think that you tend to follow a lead. They are teaching new dogs old tricks, if you follow what I mean. You tend to watch those that you want to watch and try and take on bits of how they are. But you know there are old school people that you don’t want to be like, so you tend to avoid them.” (Belinda, P6) |
| Finding64 | Service and sacrifice (core/shared dispositions) (Unequivocal) |
| Illustration | “We have been so busy over the last month. Sat here talking to you now is the longest that I have sat down in this place. I have worked 14¼ hours on two occasions and I had a 10-minutebreak in the middle of the day one day and that was it. You enjoy what you are doing but when you think about it, you think, ‘well that should not be happening” (Diane, P6-7)  “I want to show them that I can cope. I would probably take more patients than I have time for really because I want them to think that I can cope with it.” (Faye, P7) |
| Finding65 | Being with the woman and making a difference (new ways of thinking) (Unequivocal) |
| Illustration | “Just looking after laboring women, giving them support. Just being able to support them and thinking that I have made a difference. Umm y it’s not just the normal deliveries, it’s looking after the women as well. But you cannot beat the buzz of a normal delivery.” (Ann, P7)  “I like to get this relationship going and I find that then you get to know them. As you get to know them, you get to know what their problems are as well. You kind a get to know their personality and what they might be thinking and feeling and what THEY want I love that, it is really great. If you get such rapport with them, you feel like you have really been ‘with woman’. You really have been the midwife. You have done virtually everything. You have been there y even some who have abnormal deliveries, you are there all the time supporting them. And when they say, ‘you were so great’, it just makes you feel good that you have been able to make a difference.” (Faye, P7)  “My frustration is mainly to do with the women not getting the care that maybe they expected or I expected them to get.” (Belinda, P7)  “In reality it is very abnormal. And you do have to sometimes lie to keep things normal. You might say somebody is an anterior lip when they are fully just because you want to give them some extra time.” (Faye, P7) |
| **Fenwick, J et al. (2012)** | |
| Finding66 | A hectic, chaotic and “incredibly busy” environment (Unequivocal) |
| Illustration | In postnatal wards, participants said they regularly cared for six or more women and their babies at any one time. In a similar manner, antenatal clinics were described as being constantly ‘jammed packed’. Poor ‘staffing problems, bed problems, high patient loads and skill mix ‘exacerbated the situation. Working in labor ward, whilst not attracting the same comments in terms of sheer physical work load, did exact a similar toll in terms of mental or emotional work. Many participants described the labor ward as a busy, high-risk environment where normal birth was a rare event and “getting a baby into the world alive was what everyone worried about’” (Lucy, P3). |
| Finding67 | The perception and experience that communication patterns and support were poor and/or lacking. (Unequivocal) |
| Illustration | “Do you know in my whole year as a new grad [graduate] I don’t think I worked with an [midwifery] educator once.” (Anna, P3) |
| Finding68 | This the result of a system that prioritized the ‘management of complaints’ rather than care of the woman and her family. (Unequivocal) |
| Illustration | “Midwifery practice requires me to actually give more loyalty to the hospital and do all the tasks that they expect of me in a day to save[them] from being sued or just to say, ‘These jobs have been done’.” (Nell, P5) |
| Finding69 | Some hospital environments was considered to be hierarchical in nature and one where there was an obvious ‘pecking order’. (Unequivocal) |
| Illustration | “Midwifery is a hierarchical system. It is based on midwifery-in-charge [and] also who has been here the longest or who has the most experience and it’s like you were in a food chain’.” (Madison, P5) |
| Finding70 | Relationships with midwifery colleagues (Unequivocal) |
| Illustration | “It’s like how would you describe high school... it’s the same things, just the exclusion, belittlement, changeable be haviour so you never know where you stand, rolling eyes, sighing, just the hesitation before helping or the slight delay, the tone of voice... all those passive aggressive behaviors.” (Olivia, P6) |
| Finding71 | Gaining confidence and competence (Unequivocal) |
| Illustration | “I just felt like I had regained some of my confidence, that I was open to learning... I was learning midwifery. I think it is more about confidence in my support systems than anything else. Confident that I know if I am unsure that I can call, day or night.” (Anna, P6)  “I was much less scared working in caseload … you are more supported; you learn more... I felt safe because I knew the woman... I know who she is, what she wants, what her family is about. There’s much less chance that there’s something I’ve missed or that I haven’t noticed about her that I might need as part of her picture.... and if I was ever not sure about something I would always ask for a review.”(Nell,P6) |
| Finding72 | Losing confidence and struggling both personally and professionally (Unequivocal) |
| Illustration | “I just feel like I don’t have the confidence to advocate for women the way we should” (Kate, P7).  “My poor adrenals[glands] were just going full speed for that year. [I was] constantly nervous.” (Kate, P7)  “My heart would be racing, like please don’t anything go wrong, please help me get through the day”. (Belinda, P7) |
| **Clements, V et al. (2012)** | |
| Finding73 | Clinical rotations: difficult when the plan was changed. (Unequivocal) |
| Illustration | “You get pulled out to work when other areas are short. . . there is never any notice that you will not be in the area you expected…[It] can be very stressful as [there is] no warning or time to prepare for the situation. . . transitional midwives get used in this way all the time as they are the ones that can work in all areas. . .our needs are secondary to the needs of the other staff and the student midwives.” (Gladys, P4)  “I had minimal time on the delivery suite. . .didn’t get many birth as they went to the students. It would have been better to have more even distribution of time in each area. . .I need more experience in birthing.” (Xena, P4) |
| Finding74 | Supernumerary time: highly valued but not always available. (Unequivocal) |
| Illustration | “(I) had no supernumerary time. . .even though we were promised supernumerary time and an orientation in every area…but (I was) just left to get on with it…(I) was just counted in the numbers…I don’t know why, probably staffing problems. . .but it just wasn’t fair. . .it was really stressful, (and I) thought I didn’t want to carry on but I’m OK now.” (Una, P5) |
| Finding75 | Study days: a popular program element and an opportunity to connect. (Unequivocal) |
| Illustration | “If something didn’t go perfectly everyone can talk about it. . .that way we all learn from the experience.’’ (Carla, P5).  “You can talk about anything you need to. . .you don’t feel stupid…you don’t feel judged’’. (Sam, P5) |
| Finding76 | Midwife to midwife support: the importance of relationships with colleagues. (Unequivocal) |
| Illustration | “having someone to go to with a problem, who has the time to spend with you to work through and discuss with you.” (Kim, P5) |
| Finding77 | Midwifery managers: mixed messages and workloads. (Unequivocal) |
| Illustration | “After three months I was left in charge of the ward as the only midwife and when I questioned it I was told (by the man-ager) ‘Oh, you can manage an area because you’ve got experience as a nurse’. . .I can manage an area. . .but I don’t have the midwifery knowledge and skills that I feel I should have to be in charge.” (Mandy, P5) |
| Finding78 | Midwifery educators: essential but in demand. (Unequivocal) |
| Illustration | “It is there if you want it but not routinely in place: you have to ask”. (Mina, P5) |
| Finding79 | Experienced midwives: a potential lifeline. (Unequivocal) |
| Illustration | “(To) feel like I belong. . .like I can ask for help like the other(experienced) midwives without them (experienced midwives) thinking ‘Oh, there she goes again asking something stupid.” (Bernie, P6). |
| **van der Putten, Deirdre (2008)** | |
| Finding80 | Reality shock. (Unequivocal) |
| Illustration | “I was delighted to be finished. But it was really nerve-wracking … really daunting going in there … I think the labor ward particularly, it’s a big challenge, and you ’re terrified in case you ’ll miss something.” (Anne, P3)  “From the start, you were signing your own discharges as a staff midwife, and then you had to sign up your student’s discharges too. So your name was on these and you know, that was a reality check ... My name is on this now ... They ’ll be looking for me. Not my senior person – me – it was very stressful and with so much litigation now you know, it’s difficult.” (Clare, P3) |
| Finding81 | Living up to expectations (Unequivocal) |
| Illustration | “It’s such a challenge really … the women are all so well up these days and they all expect so much, you know, and trust you implicitly … everything just has to be perfect …it can be very stressful and you ’re drained by the end of the shift.” (Anne, P3)  “…some days it’s just so busy, you might have thirty women and babies, that’s sixty people, and there might ’t be many of you on and the women expect such attention especially with the breastfeeding…and they ’re used to the one-to-one care they ’ve had in the labor ward… and on the postnatal it’s just so short staffed … we just don ’t have the time to give that level of care, even though you ’d like to.” (Diane, P4) |
| Finding82 | Theory–practice gap (Unequivocal) |
| Illustration | “…yeah, you realize, that’s not what they told us in the classroom ... they went on about woman centered care and empowering the women to make the decisions about their labor themselves but…you know spontaneous rupture of membranes is the way to go yet nine out of ten times it’s ARM and ... the examinations … VEs every two hours … there’s no method behind that.” (Diane, P4)  “it’s very difficult…we learnt all about midwifery models of care and being woman-centered, that that’s the best way, but you know once they ’re in the hospital it can be so different ... unless they ’re really assertive women they [midwives] sort of actively manage the labor the whole time …like most Irish hospital.” (Eithne, P4) |
| Finding83 | Clinical support and mentorship (Unequivocal) |
| Illustration | “…but also being able to put your hand up and ask for help if you needed it on the wards … I feel very lucky that they have looked after me and have given me great support.” (Barbara, P4)  “... they orientate you, they explain everything to you, the routine, the procedures and practices – and they still keep an eye on you – you know make sure you ’re doing ok and that gives you confidence.” (Barbara, P4) |
| Finding84 | Continuous professional education (Unequivocal) |
| Illustration | Participants reported (n=4) that their new level of responsibility stimulated an awareness of the importance of continuous professional education (CPE) in order to continue to provide safe care to women and was often triggered by reflection following an uncomfortable event. (P4) |

**Supplementary Table 3.**

**Summary of study finding, categories, and synthesized categories to generate synthesized findings on experiences of newly qualified midwives during their transition to practice.**

| **Synthesized finding 1: Multi-dimensional challenges** | | | |
| --- | --- | --- | --- |
| Findings  (Credibility Rating) | | Categories | Synthesized  Category |
| 5 | Labor ward viewed as a negative working environment (Unequivocal) | Shock from a realistic clinical settings | Multi-dimensional challenges |
| 11 | Inadequate resources (Unequivocal) |  |  |
| 17 | Too busy (Unequivocal) |  |  |
| 32 | High workload (Unequivocal) |  |  |
| 48 | Organizational tasks (Unequivocal) |  |  |
| 58 | High risk, high intensity, high stress (Unequivocal) |  |  |
| 66 | A hectic, chaotic and “incredibly busy” environment (Unequivocal) |  |  |
| 80 | Reality shock. (Unequivocal) |  |  |
| 36 | Job insecurity (Unequivocal) |  |  |
| 40 | Employment conditions (Unequivocal) |  |  |
| 43 | Working as locum (Equivocal) |  |  |
| 9 | Theory practice gap (Unequivocal) |  |  |
| 60 | There is a gap between the actual work and the ideal. (Unequivocal) |  |  |
| 63 | Old school midwives (entrenched viewpoint) (Unequivocal) |  |  |
| 82 | Theory–practice gap (Unequivocal) |  |  |
| 1 | Expectations of self (Unequivocal) | High expectations of themselves and from others |  |
| 15 | Set high standards for yourself (Equivocal) |  |  |
| 42 | Personal demands (Unequivocal) |  |  |
| 51 | Personal demands (Unequivocal) |  |  |
| 2 | Expectations of others (Unequivocal) |  |  |
| 6 | Supervisory and teaching roles and responsibilities of newly qualified midwives (Unequivocal) |  |  |
| 13 | Workplace conflict (Unequivocal) |  |  |
| 16 | Attention and evaluation from colleagues (Unequivocal) |  |  |
| 64 | Service and sacrifice (core/shared dispositions) (Unequivocal) |  |  |
| 77 | Midwifery managers: mixed messages and workloads. (Unequivocal) |  |  |
| 81 | Living up to expectations (Unequivocal) |  |  |
| 12 | Lack of transitioning support system (Unequivocal) | Lack of transitional support |  |
| 25 | Graduates described midwifery units as being non-conducive for effective transition. (Unequivocal) |  |  |
| 33 | Becoming a team member (Unequivocal) |  |  |
| 46 | Working in different regions was mentally demanding for NQMs. (Equivocal) |  |  |
| 67 | The perception and experience that communication patterns and support were poor and/or lacking. (Unequivocal) |  |  |
| 73 | Clinical rotations: difficult when the plan was changed. (Unequivocal) |  |  |
| 8 | Negative attitudes of experienced midwives (Unequivocal) |  |  |
| 24 | Newly qualified midwives expressed dissatisfaction about the mentoring process. (Unequivocal) |  |  |
| 54 | Cope was admired by colleagues and asking for help was a weakness. (Unequivocal) |  |  |
| 70 | Relationships with midwifery colleagues (Unequivocal) |  |  |
| **Synthesized finding 2: Physical and emotional responses** | | | |
| Findings  (Credibility Rating) | | Categories | Synthesized  Category |
| 26 | Physical exhaustion (Unequivocal) | Physical fatigue | Physical and emotional responses |
| 29 | Sleep deficiency (Unequivocal) |  |  |
| 10 | Lack of confidence and skills (Unequivocal) | Negative feelings, fear and loneliness, and lack of confidence |  |
| 14 | Feeling challenged (Unequivocal) |  |  |
| 53 | Uncertainty and considerable challenge. (Unequivocal) |  |  |
| 72 | Losing confidence and struggling both personally and professionally (Unequivocal) |  |  |
| 7 | Administrative roles of newly qualified midwives (Unequivocal) |  |  |
| 27 | Emotional exhaustion (Unequivocal) |  |  |
| 28 | Mental exhaustion (Unequivocal) |  |  |
| 30 | Work–Life balance (Unequivocal) |  |  |
| 31 | No time outside of work (Unequivocal) |  |  |
| 44 | Balancing work private life (Unequivocal) |  |  |
| 52 | Feel traumatized and angry (Unequivocal) |  |  |
| 74 | Supernumerary time: highly valued but not always available. (Unequivocal) |  |  |
| **Synthesized finding 3: Demands and expectations** | | | |
| Findings  (Credibility Rating) | | Categories | Synthesized  Category |
| 3 | Accessible support (Unequivocal) | Support from peers, colleagues and managers | Demands and expectations |
| 4 | Peer support (Unequivocal) |  |  |
| 21 | The need for support from an approachable colleague in the clinical area (Unequivocal) |  |  |
| 23 | Team-building events would be beneficial (Equivocal) |  |  |
| 37 | Working in a team (Unequivocal) |  |  |
| 45 | Colleagues were mentioned as both a demand and a resource. (Unequivocal) |  |  |
| 55 | Relationships with colleagues (Unequivocal) |  |  |
| 76 | Midwife to midwife support: the importance of relationships with colleagues. (Unequivocal) |  |  |
| 78 | Midwifery educators: essential but in demand. (Unequivocal) |  |  |
| 79 | Experienced midwives: a potential lifeline. (Unequivocal) |  |  |
| 83 | Clinical support and mentorship (Unequivocal) |  |  |
| 18 | Disappointment (Unequivocal) | Improve professional competence |  |
| 35 | Providing care for women (Unequivocal) |  |  |
| 59 | Full of joy of rebirth, a sense of accomplishment and value (Unequivocal) |  |  |
| 38 | Working with women (Unequivocal) |  |  |
| 49 | “Contact with clients” as particularly motivating. (Unequivocal) |  |  |
| 56 | Relationships with women (Unequivocal) |  |  |
| 65 | Being with the woman and making a difference (new ways of thinking) (Unequivocal) |  |  |
| 41 | Personal resources (Unequivocal) |  |  |
| 47 | Working autonomously was identified by participants as both a job demand and a job resource. (Unequivocal) |  |  |
| 50 | Personal resources (Unequivocal) |  |  |
| 57 | A new beginning (Unequivocal) |  |  |
| 20 | Transitioning to practice in a familiar environment eased their transition. (Unequivocal) |  |  |
| 19 | Getting stuck in learn from mistakes gaining confidence (Unequivocal) |  |  |
| 22 | Additional training needs (Equivocal) |  |  |
| 34 | Additional midwifery skills and procedures (Unequivocal) |  |  |
| 39 | Variety of the work (Unequivocal) |  |  |
| 61 | Look forward to diversified career development (Unequivocal) |  |  |
| 71 | Gaining confidence and competence (Unequivocal) |  |  |
| 75 | Study days: a popular program element and an opportunity to connect. (Unequivocal) |  |  |
| 84 | Continuous professional education (Unequivocal) |  |  |
| 62 | See the development potential of the profession and desire to standardize the industry management. (Unequivocal) | Standardize the management system |  |
| 68 | This the result of a system that prioritized the ‘management of complaints’ rather than care of the woman and her family. (Unequivocal) |  |  |
| 69 | Some hospital environments was considered to be hierarchical in nature and one where there was an obvious ‘pecking order’. (Unequivocal) |  |  |
